# Age-Related Increases in IGFBP2 Increase Melanoma Cell Invasion and Lipid Synthesis

**DOI:** 10.1158/2767-9764.CRC-23-0176

**Published:** 2024-08-02

**Authors:** Gretchen M. Alicea, Payal Patel, Marie E. Portuallo, Mitchell E. Fane, Meihan Wei, Yash Chhabra, Agrani Dixit, Alexis E. Carey, Vania Wang, Murilo R. Rocha, Reeti Behera, David W. Speicher, Hsin-Yao Tang, Andrew V. Kossenkov, Vito W. Rebecca, Denis Wirtz, Ashani T. Weeraratna

**Affiliations:** 1 Department of Biochemistry and Molecular Biology, Johns Hopkins Bloomberg School of Public Health, Baltimore, Maryland.; 2 Institute for Nanobiotechnology, Johns Hopkins University, Baltimore, Maryland.; 3 Department of Oncology, Sidney Kimmel Comprehensive Cancer Center, Johns Hopkins University School of Medicine, Baltimore, Maryland.; 4 The Wistar Institute, Philadelphia, Pennsylvania.; 5 The Fox Chase Cancer Center, Philadelphia, Pennsylvania.; 6 Department of Chemical and Biomolecular Engineering, Johns Hopkins University, Baltimore, Maryland.

## Abstract

Aged patients with melanoma (>65 years old) have more aggressive disease relative to young patients (<55 years old) for reasons that are not completely understood. Analysis of the young and aged secretome from human dermal fibroblasts identified >5-fold levels of IGF-binding protein 2 (IGFBP2) in the aged fibroblast secretome. IGFBP2 functionally triggers upregulation of the PI3K-dependent fatty acid biosynthesis program in melanoma cells. Melanoma cells co-cultured with aged dermal fibroblasts have higher levels of lipids relative to those co-cultured with young dermal fibroblasts, which can be lowered by silencing IGFBP2 expression in fibroblasts prior to treating with conditioned media. Conversely, ectopically treating melanoma cells with recombinant IGFBP2 in the presence of conditioned media from young fibroblasts or overexpressing IGFBP2 in melanoma cells promoted lipid synthesis and accumulation in melanoma cells. Treatment of young mice with rIGFBP2 increases tumor growth. Neutralizing IGFBP2 *in vitro* reduces migration and invasion in melanoma cells, and *in vivo* studies demonstrate that neutralizing IGFBP2 in syngeneic aged mice reduces tumor growth and metastasis. Our results suggest that aged dermal fibroblasts increase melanoma cell aggressiveness through increased secretion of IGFBP2, stressing the importance of considering age when designing studies and treatment.

**Significance:**

The aged microenvironment drives metastasis in melanoma cells. This study reports that IGFBP2 secretion by aged fibroblasts induces lipid accumulation in melanoma cells, driving an increase in tumor invasiveness. Neutralizing IGFBP2 decreases melanoma tumor growth and metastasis.

## Introduction

Melanoma is the deadliest form of skin cancer. Despite the advances in therapy over the past several years, melanoma remains a deadly disease due to its high metastatic potential (1, 2). Older patients with melanoma display a more aggressive disease with a higher rate of metastasis to distant organs and increased resistance to targeted therapy (3). Recent evidence from our laboratory shows that factors secreted by aged fibroblasts can drive changes in the way melanoma cells invade, metastasize, and respond to therapy (4–7). These included secreted proteins and molecules such as lipids (e.g., ceramides; ref. 5). Metabolism serves a key role in melanoma therapy escape as well as metastasis (8, 9), and we have shown that melanoma cells take up lipids through age-related expression of the SLC27A2 solute carrier family 27 member 2 also known as fatty acid transporter FATP2 in response to ceramides produced by aged fibroblasts, which are then utilized to escape therapy (5). A study by Zhang and colleagues (10) has also shown that another member of the SLC27A family, SLC27A1 also known as FATP1, could be induced by adipocytes in melanoma cells, allowing them to take up lipids from those adipocytes and become more metastatic. Additionally, increased lipids in cancer cells are a marker of aggressive tumors, and increases in lipogenic enzymes, such as acetyl-CoA carboxylase, fatty acid synthase (FASN), and ATP citrate lyase, are found in almost all advanced tumors (11–14).

To determine whether aged fibroblasts could induce lipid synthesis and uptake in melanoma cells, we examined secreted factors in aged fibroblasts identified as regulators of lipid synthesis. Of these, the Insulin Like Growth Factor-binding protein 2 (IGFBP2) was the most interesting candidate as high expression of IGFBP2 correlates with increased mortality (15) and is involved with FASN. IGFBP2 is part of a family of six IGF-binding proteins and the second most abundant IGF family member in humans (16, 17). IGFBP2 has been linked with several aging diseases, such as diabetes, insulin resistance, and progeria (accelerated aging; refs. 18, 19). High IGFBP2 expression protects against type II diabetes, regulates glucose metabolism, and increases during aging and even more during premature aging (19, 20). IGFBP2 specifically can bind to insulin, IGF-1, and IGF-2 but binds with the highest affinity to IGF-2; however, it plays multiple roles in cancer in an IGF-independent manner. High levels of IGFBP2 correlate with proliferation, epithelial-to-mesenchymal transition, invasion, and reduction of cell death in glioblastoma, prostate cancer, breast cancer, and melanoma (16, 21–23). Indeed, Li and colleagues (24) suggest that highly expressed IGFBP2 acts as a hub for multiple signaling pathways.

In melanoma, IGFBP2 plays additional roles in the activation of the Epidermal Growth Factor Receptor (EGFR)-Signal Transducer adn Activator of Transcripton (STAT3) pathway, activating Programmed Death Ligand 1 (PDL1), and contributing to angiogenesis (24). Furthermore, high levels of IGFBP2 correlate with resistance to Mitogen Activated Protein Kinase (MAPK) inhibitors and may represent a marker for therapy resistance in melanoma (25). In glioblastoma and prostate cancer, IGFBP2 expression is negatively regulated by Phosphatase and Tensin homolog (PTEN) and positively correlated with AKR mouse thymoma kinase (AKT) expression (26). Loss of PTEN in melanoma may therefore increase levels of IGFBP2 and, we hypothesize, impact the standard of care (BRAF/MEK inhibition) for melanoma cells. Finally, IGFBP2 is also a component of the senescence-associated secretory phenotype associated with increased aggressiveness in cancer, and senescence and aging are often linked (27–29). In the current study, we explore the expression of IGFBP2 in melanoma cells in an aged versus young microenvironment and its subsequent impact. Taken together with our previously published work, these data add a new dimension, showing that aged fibroblasts can drive melanoma cells to not only take up lipids but also synthesize *de novo* lipids through pathways driven by IGFBP2.

## Materials and Methods

### Cell culture

1205 Lu, WM164, WM793, and WM9 cells were maintained in MCDB153 (Sigma)/L-15 (CellGro; 4:1 ratio) supplemented with 2% FBS and 1.6 mmol/L CaCl_2_ (tumor growth media). WM983b cells were maintained in DMEM supplemented with 10% FBS. YUMM1.7 murine cultured melanoma cells were maintained in DMEM F-12 (HEPES/glutamine) supplemented with 10% FBS and 100 units per mL penicillin and streptomycin. Dermal fibroblast cell lines were obtained from Coriell Institute for Medical Research Biobank. Fibroblasts were maintained in DMEM supplemented with 10% FBS. Cell lines were cultured at 37°C in 5% CO_2_, and the medium was replaced as required. Cell stocks were fingerprinted using the AmpFLSTR Identifiler PCR Amplification Kit from Life Technologies TM at the Wistar Institute Genomics Facility. Although it is desirable to compare the profile with the tissue or patient of origin, our cell lines were established over the course of 40 years, long before acquisition of normal control DNA was routinely performed. However, each short tandem repeat profile is compared with our internal database of more than 200 melanoma cell lines and control lines, such as HeLa and 293T. Cell culture supernatants were tested for mycoplasma using a Lonza MycoAlert assay at the University of Pennsylvania Cell Center Services.

### Heatmap analysis

The list of 91 significantly differentially secreted proteins between aged and young fibroblasts was obtained from Kaur and colleagues (30) and re-analyzed using ingenuity pathway analysis for enrichment of specific functions and diseases. Enrichments with at least 10 affected proteins that pass the *P* value <10^−5^ threshold were considered significant. Individual non-cell-type or cancer-specific functions and diseases were combined into common categories and reported along with the total category number of unique proteins and minimal *P* value. These data are available upon request.

### Western blot

Cell lines were plated and collected with RIPA buffer. Total protein lysate was quantified using a Pierce BCA assay kit (# 23225, Thermo Fisher Scientific), and 25 μg of protein was prepared in sample buffer, boiled, and loaded into NuPAGE 4% to 12% Bis-Tris protein gels (#NP0321BOX, Thermo Fisher Scientific) and run at 160 V. Proteins were then transferred onto a polyvinylidene difluoride membrane using the iBlot system (Invitrogen) and blocked in 5% milk/TBS + polysorbate 20 (TBST) for 1 hour. Primary antibodies were diluted in 5% milk/TBST and incubated at 4°C overnight. The membranes were washed in TBST and probed with the corresponding horseradish peroxidase–conjugated secondary antibody at 0.2 μg/ml. Proteins were visualized using ECL prime (Amersham, Uppsala, Sweden) and detected using ImageQuant LAS 4000 (GE Healthcare Life Sciences, Pittsburgh, PA).

### Antibodies

Antibodies were purchased from the following commercial vendors and used in the following dilutions for Western blot: GAPDH (1:10,000, Cell Signaling Technology 2118S), HSP90 (1:10,000, Cell Signaling Technology 4877S), IGFBP2 (1:1,000, Cell Signaling Technology 3922), p-AKT Ser473 (1:1,000, Cell Signaling Technology 4060), T-AKT (1:2,000, Cell Signaling Technology 2920), FASN, (1:1,000, Cell Signaling Technology 3180S).

### Matrigel invasion assay

Matrigel-coated (invasion) 8 μm pore size translucent 24-well plate transwell chambers (BD Biosciences, San Jose, CA, USA) were used to evaluate the migration and invasion potential of melanoma cells cultured in different conditions. Briefly, 500 μL of growth medium (20% FBS) was added to the bottom of each well, and a total of 2.5 × 10^4^ cells resuspended in 250 μL of young conditioned media (CM) in the presence or absence of rIGFBP2 (150 ng/mL; 674-B2-025 from R&D Systems) or aged CM in the presence or absence of 5 mg/mL neutralizing IGFBP2 antibody (AF674 from R&D Systems) were seeded on top. After 18 hours of incubation at 37°C, 5% CO_2_, non-invading cells were removed by wiping the upper side of the membrane, and invading cells fixed with methanol and stained with crystal violet (Sigma-Aldrich, St. Louis, MO, USA).

### Wound healing assay

Melanoma cells were plated to 90% confluency, and wells were scratched using a sealed pipette tip. Melanoma cells were then cultured with young CM in the presence or absence of (150 ng/mL) rIGFBP2 (674-B2-025 from R&D Systems) and aged CM from fibroblasts treated with neutralizing (5 mg/mL) IGFBP2 antibody (AF674 from R&D Systems) for 24 hours. Wells were imaged using a Nikon TE2000 inverted microscope.

### 3D spheroids

A total of 5,000 melanoma cells were plated in 1.5% agar, and spheroids were allowed to form for 3 days. Spheroids were then embedded in rat-tail collagen at 1.5 mg/mL (Life Technologies). Spheroids were then cultured with unconditioned media, young CM, and aged CM in the presence or absence of rIGFBP2 or neutralizing IGFBP2 or shIGFBP2 media from aged fibroblasts. Spheroid invasion was imaged with a Nikon TE2000 inverted microscope.

### Immunohistochemistry

Mouse tumor and lung were paraffin-embedded and sectioned. Paraffin-embedded sections were rehydrated through a series of xylene and different concentrations of alcohol, which was followed with a rinse in water and washed in PBS. Slides were coated with an antigen retrieval buffer (#3300, Vector Labs, Burlingame, CA) and steamed for 20 minutes. The slides were then blocked in a peroxide blocking buffer (#TA060H2O2Q, Thermo Fisher Scientific) for 15 minutes, followed by protein block (#TA-060-UB, Thermo Fisher Scientific) for 5 minutes, and incubated with the primary antibody of interest which was prepared in antibody diluent (S0809, Dako). The slides were put in a humidified chamber at 4°C overnight. The slides were then washed with PBS and incubated in biotinylated anti-rabbit (#ab64256 Abcam), followed by streptavidin–horseradish peroxidase solution at room temperature for 20 minutes (#TS-060-HR Thermo Fisher Scientific). Samples were then washed with PBS and incubated with 3-amino-9ethyl-I-carboazole) chromogen for the appropriate amount of time after optimization (#TA060SA, Thermo Fisher Scientific). Slides were then washed with water and incubated in Mayer’s hematoxylin (MHS1, Sigma) for 1 minute, rinsed with water, and mounted in Aquamount (#143905, Thermo Fisher Scientific). Lungs were assessed for localization of mCherry-positive melanoma cells using a Nikon TE2000 inverted microscope and quantitated using QuPath.

### Immunofluorescence

Samples were fixed with 4% paraformaldehyde for 15 minutes at room temperature. After the samples were washed with PBS, they were incubated with BODIPY 493/503 or BODIPY 505/515 (1:3,000, Thermo Fisher Scientific) for 15 minutes at room temperature. The samples were then washed with PBS and stained with DAPI (Invitrogen, 1:5,000) for 5 minutes. After the samples were washed with PBS, they were mounted in ProLong Gold antifade reagent. Lipid droplet (BODIPY stain) intensity was quantified with Adobe Photoshop software. Channels were separated, and melanoma cell staining intensity was quantified across different culture conditions. Values were then compared between conditions using an unpaired *t* test.

### Oil Red O staining

Tumor tissue was embedded in Tissue-Tek Optimal Cutting Temperature (OCT) Compound and sectioned. Frozen sections were fixed in 10% formalin for 10 minutes and then briefly washed with water for 1 minute. Next, sections were rinsed with 60% isopropanol for approximately 5 minutes followed by staining with filtered Oil Red 0.5% in isopropanol for about 15 minutes. Subsequently, sections were rinsed with 60% isopropanol, with the solution being changed twice, each time for 5 minutes. Next, the samples were counterstained with hematoxylin, rinsed with water, and mounted in Aquamount before imaging.

### *In vivo* allograft assays

All animal experiments were approved by the Institutional Animal Care and Use Committee of the Johns Hopkins University (Protocol M019H421: Microenvironmental regulation of metastasis and therapy resistance) and were performed in an Association for the Assessment and Accreditation of Laboratory Animal Care–accredited facility. Mice were housed in a vivarium maintained at 20°C ± 2°C, 42% humidity, with a 12-hour light–dark cycle with free access to food and water. The maximum tumor size allowed under this protocol was 2,000 mm^3^. One tumor in our experiments exceeded this size because it grew unexpectedly fast after the preceding measurement (2,086 mm^3^). The young mice were utilized at 8 weeks and the aged mice at 52 weeks (Charles River Laboratories). Aged male mice were single-housed, and young male mice were housed in groups of no more than five per cage. YUMM1.7 mCherry murine melanoma cells (2.5 × 10^5^) were injected subdermally into aged (52 weeks old) and young (6–8 weeks old) C57/BL6 mice (Charles River Laboratories). Data collection and analysis were conducted without blinding to the experimental conditions. Mice were randomly assigned to treatment groups, and the significance of tumor growth was assessed using ANOVA analysis. Tumors were allowed to grow, and treatment with rIgfbp2 (674-B2-025 from R&D Systems), Igfbp2-neutralizing antibody (AF674 from R&D Systems), or IgG control (AB-105-C from R&D Systems) was administered. rIGFBP2 experiments: young mice were injected intraperitoneally with 500 ng of rIgfbp2 in 100 μL of PBS every 2 days until tumors reach a maximum of 2,000 mm^3^. Neutralizing Igfbp2 experiments: YUMM1.7 murine melanoma cells (2.5 × 10^5^) expressing mCherry were injected subdermally into aged mice. Mice were i.p. injected with neutralizing Igfbp2 antibody (AF797 from R&D systems) or an IgG control (AB-105-C from R&D systems) at a concentration of 1 mg/kg every day until tumors reached 1,500 mm^3^. Tumor sizes for each experiment were measured every 2 days using digital calipers, and tumor volumes were calculated using the following formula: volume = 0.5 × (length × width^2^). Mice were euthanized after 5 weeks or when a group reached 1,500 mm^3^, and tumor and lung tissues were preserved. Half of the tissue was embedded in paraffin, and the other half was flash-frozen and processed for protein analysis.

### shRNA, lentiviral production, and infection

IGFBP2 shRNA was obtained from the TRC shRNA library available at the Wistar Institute. Lentiviral production was performed according to the protocol suggested by the Broad Institute. Briefly, 293T cells were at 70% confluency and were co-transfected with shRNA plasmid and the lentiviral packaging plasmids (pCMV-dR8.74psPAX2 and pMD2.G for second generation; pMDLg/pRRE, pRSV/REV, and PMD2.G for third generation). IGFBP2 overexpression vector was obtained from Dharmacon (catalog number: OHS5836-EG3485). Appropriate empty vector and scrambled controls were created for overexpressing and shRNA constructs, respectively. Cells were transduced with lentivirus for 48 hours, allowed 48 hours to recover, and then treated with appropriate antibiotic selection (puromycin) with previously established kill curves for each cell line.

### Quantitative RT-PCR

Melanoma cells were treated with young CM or aged CM, and RNA was extracted using TRIzol (Invitrogen) and the RNeasy Mini Kit (Qiagen) as protocol instructions. 1 μg RNA was used to prepare cDNA using the iScript DNA Synthesis Kit (#1708891, Bio-Rad, CA). cDNA was diluted at a 1:5 ratio before use for further reactions. Each 20 μL well reaction comprised of 10 μL Power SYBR Green Master Mix (4367659, Invitrogen), 1 μL cDNA, and 1 μL primer.

#### IGFBP2

F-5′AGCCCAAGAAGCTGCGACCAC′3

R-5′CTGCCCGTTCAGAGACATCTTGC″3

#### IGFBP3

F-5′ ATGCAGCGGGCGCGAC 3′

R- 5′ CTA​CTT​GCT​CTG​CAT​GCT​GTA​GCA 3′

#### IGFBP1

F-5′TGCTGCAGAGGCAGGGAGCCC-3′

5′-5′AGG​GAT​CCT​CTT​CCC​ATT​CCA-3

#### IGFBP4

F-5′CGCCCCCAGCAGACTTCAC-3′

R-5′CTCCTCTTTTGCACCCCTCCCATTT-3′

#### IGFBP5

F-5′AGATGCCTTCAGCAGAGTG-3′

R-5′ACATGCGCCTTGATGTCGTG-3′

#### IGFBP6

F-5′GACCAGGAAAGAATGTGAAAGTGA-3′

R-5′GCTCTGCCAATTGACTTTCCTTAG-3′

#### 18S

F-5′GAGGATGAGGTGGAACGRTGT-3′

R-5′TCT TCA GTC GCT CCA GGT CT-3′

The final concentration used was 0.5 μmol/L. Standard curves were generated for all primers, and each set of primers was normalized to an 18S primer pair acquired from IDT Technologies.

### Organotypic 3D skin reconstructs

Organotypic 3D skin reconstructs were generated by plating 6.4 × 10^4^ fibroblasts in each insert on top of the acellular layer (BD #355467 and Falcon #353092) and incubated for 45 minutes at 37°C in a 5% CO_2_ tissue culture incubator. DMEM containing 10% FBS was added to each well of the tissue culture trays and incubated for 4 days. Reconstructs were then incubated for 1 hour at 37°C in Hank’s Balanced Salt Solution containing 1% dialyzed FBS (wash media). Washing media was removed and replaced with reconstruct media. Keratinocytes (4.17 × 10^5^) and melanoma cells (8.3 × 10^4^) were added to the inside of each insert. Media was changed every other day until day 18 when reconstructs were harvested, fixed in 10% formalin, paraffin-embedded, sectioned, and stained.

### Reverse phase protein array

Proteins were isolated from YUMM1.7 mCherry tumor lysates. Reverse phase protein array (RPPA) was performed using a total of 53 antibodies. A logarithmic value was generated, reflecting the quantitation of the relative amount of each protein in each sample. Differences in relative protein loading were determined by the median protein expression for each sample across all measured proteins using data that had been normalized to the median value of each protein. The raw data were then divided by the relative-loading factor to determine load-corrected values. Logarithmic values for each protein were mean-centered to facilitate concurrent comparisons of different proteins.

### ELISA

CM from fibroblasts were collected after 48 hours in DMEM growth medium and filtered through a 0.45-μm low protein binding polyvinylidene difluoride filter. Harvested CM was normalized to the fibroblast cell count and assessed for IGFBP2 using an ELISA purchased from R&D Systems (DGB200). Assay was performed as per the manufacturer’s instructions.

### Data availability

Proteomics: Raw data for this study were generated at Proteomics Core Facility of the Wistar Institute and may not be available. Derived data were published in full in Supplementary Table S1 of Kaur and colleagues (30), and these and all other data supporting the findings of this study are available from the corresponding author upon request.

## Results

**Figure 1:**
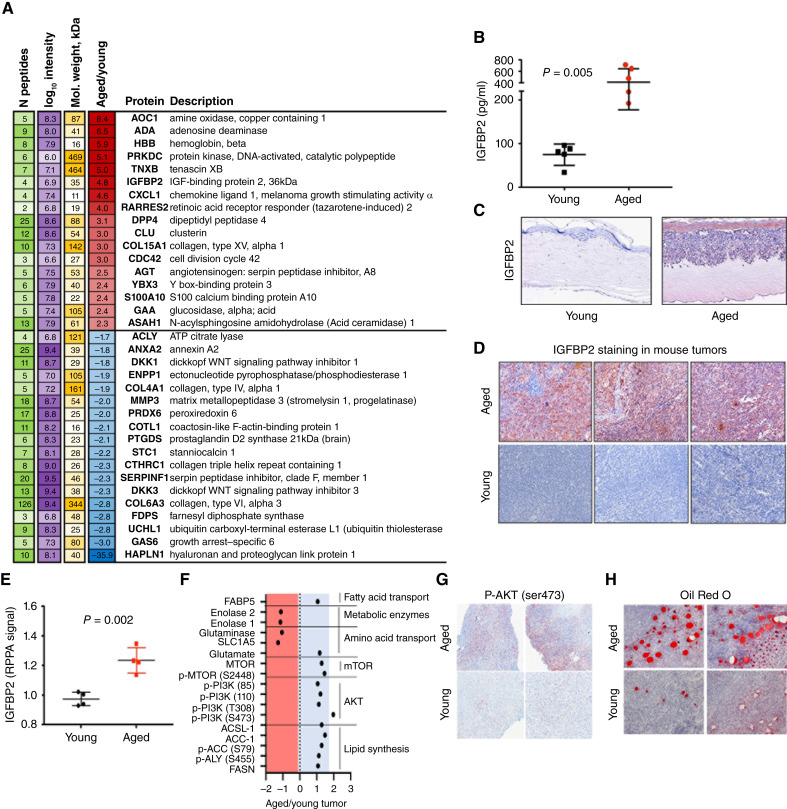
Aged fibroblasts secrete high levels of IGFBP2. **A,** Re-analysis of proteomics on CM from young and aged dermal fibroblasts, published in Kaur and colleagues (30), showing differentially expressed proteins between the two groups, in which red denotes increased expression in aged as compared with young, and blue denotes decreased expression. **B,** IGFBP2 ELISA analysis in young and aged human dermal fibroblast CM (*P* = 0.0055). **C,** IGFBP2 staining in human melanoma skin reconstructs with young or aged donor-derived dermal fibroblasts. **D,** IGFBP2 staining in primary tumor tissue from young and aged C57BL6 mice. **E,** RPPA analysis of young and aged YUMM1.7 mouse tumor lysate for IGFBP2 expression (*P* = 0.002). **F,** Pathway analysis of RPPA analysis of young and aged YUMM1.7 mouse tumor lysate. **G,** P-AKT (Ser473) staining of tumors in young and aged mice. **H,** Oil Red O staining of tumors in young and aged mice.

We revisited previously published secretomic data from dermal fibroblasts from normal healthy human donors obtained from Coriell and derived from the Baltimore Longitudinal Study of Aging that showed 91 proteins significantly differentially secreted (FDR < 1%) between aged and young fibroblasts (30). The list of proteins was analyzed for enrichment of specific functions and diseases using ingenuity pathway analysis. Significantly enriched categories (*P* value < 10^−5^) are shown in Fig. 1A. IGFBP2 was one of the proteins that displayed increased expression in aged fibroblasts from donors aged 55 and over when compared with young fibroblasts aged 35 and younger (Fig. 1A). While not the most highly increased protein on the list, IGFBP2 is of particular interest in aging and cancer. IGFBPs have been shown to be secreted during senescence to a greater extent, and their high expression has been linked with metabolic dysregulation such as diabetes, obesity, and insulin resistance (31, 32). Also, IGFBP2 is known to interact with IGFs, which are involved in distinct processes such as lipid and fatty acid synthesis and aging, strengthening our rationale for exploring IGFBP2 further (16, 29).

We analyzed IGFBP2 expression by ELISA analysis of the supernatant (CM, 48 hours) of young (<35 years) and aged (>55 years) human dermal fibroblasts grown in serum-free media (Fig. 1B), and found it to be increased. Furthermore, we checked the mRNA levels of all members of the IGFBP family in the young and aged dermal fibroblasts, and only *IGFBP2* was upregulated in the aged fibroblasts compared with the young fibroblasts (Supplementary Fig. S1A). Using the Human Protein Atlas, we found that older patients have a trend of higher expression of *IGFBP2* compared with young patients (Supplementary Fig. S1B). When stratified by *IGFBP2* expression in aged and young patients, we observed that this was pronounced in the aged population, in which aged patients with low *IGFBP2 *expression have a higher overall survival probability versus those with high *IGFBP2* expression (Supplementary Fig. S1C).

Since data in the human protein atlas indicated that older patients with melanoma with high IGFBP2 expression have lower probability of survival, we wanted to determine if IGFBP2 secreted from aged fibroblasts could have an impact on melanoma cells. We built skin reconstructs, an artificial 3D representation of human skin, as previously described (33). Skin reconstructs were built with either young or aged fibroblasts and identical human melanoma cell lines. We have previously shown that melanoma cells behave very differently in aged skin reconstructs, growing and invading more rapidly than when they are built with young fibroblasts (4). When built into reconstructs with aged fibroblasts, melanoma cells increased the expression of IGFBP2 (Fig. 1C; Supplementary Fig. S1D).

To ascertain if this result could be validated *in vivo*, we injected YUMM1.7 melanoma cells derived from the *B-Raf*^*V600E*^/*Pten*^-/-^/*Cdkn2A*^-/-^ mouse model intradermally into C57BL/6 mice of either 8 weeks of age or >52 weeks of age. We stained the subsequent tumors with Igfbp2 antibody and found that the tumors from the aged mice stained more intensely for Igfbp2 than those from young mice (Fig. 1D). RPPA of the tumor lysates also confirmed increased Igfbp2 in melanoma tumors borne in aged mice (Fig. 1E), as well as an increase in fatty acid synthesis proteins, p-AKT, and the mTOR pathway (Fig. 1F). IHC analysis of aged mice displayed significantly higher levels of p-AKT^Ser473^ than young mice (Fig. 1G; Supplementary Fig. S1E), and Oil Red O staining of adipocytes was also increased in aged compared with young mouse tumors, supporting the *in vitro* observations (Fig. 1H; Supplementary Fig. S1F). Overall, our results are consistent with increases in Igfbp2 in fibroblasts during aging and correspondingly in tumors in an aged microenvironment.

**Figure 2:**
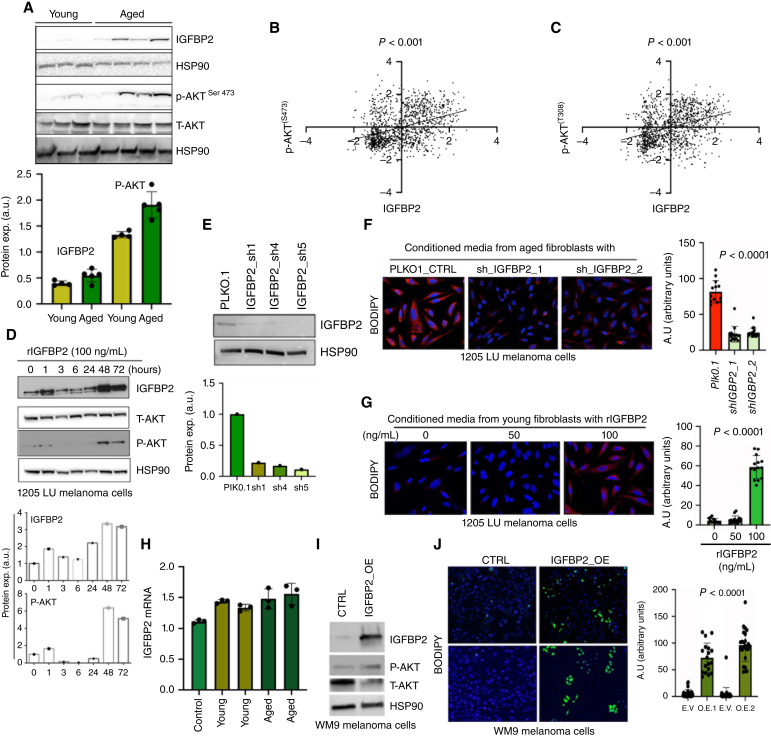
IGFBP2 induces fatty acid synthesis in melanoma cells. **A,** Western blot analysis of Igfbp2, phospho-Akt, total Akt and Hsp90 (loading control) from young and aged tumor lysate. Quantification of immunoblotting of Igfbp2 and phospho-Akt relative to Hsp90 loading control. **B,** Correlation analysis of IGFBP2 protein expression and p-AKT S473 expression from RPPA of melanoma PDX samples. **C,** Correlation analysis of IGFBP2 protein expression and p-AKT T308 expression from RPPA of melanoma PDX samples. **D,** Western blot analysis of melanoma cells (1205Lu) treated with recombinant IGFBP2 (100 ng/mL) at different times. Cells were probed for IGFBP2, phospho-AKT, total AKT, and HSP90 (loading control). **E,** Western blot analysis of IGFBP2 knockdown in aged fibroblasts. **F,** BODIPY (505/515) staining of human melanoma cells (1205Lu) cultured with aged fibroblast CM from fibroblasts transduced with empty vector (PLKO.1) or shRNA constructs (shIGFBP2). Quantification of BODIPY. **G,** BODIPY (505/515) staining of melanoma cells treated with CM from young fibroblasts treated with recombinant IGFBP2 (50 and 100 ng/mL). Quantification of BODIPY. **H,** RT-PCR of IGFBP2 expression in melanoma cells after treatment with young or aged CM. **I,** Western blot analysis of IGFBP2, P-AKT, total AKT, and HSP90 in melanoma cells transfected with either an empty vector control (CTRL) or IGFBP2 (IGFBP2 OE). **J,** BODIPY (493/503) staining of melanoma cells after transfection with either control or IGFBP2 constructs with quantification. GraphPad Prism 8 was used for plotting graphs and statistical analysis. Unpaired *t* test was performed.

As we observed an increase in signaling through the Akt and Mtor pathways in the RPPA data, we wanted to confirm that increased Igfbp2 altered these signaling pathways in melanoma. We analyzed the tumor lysates from young and aged animals and observed an increase in both Igfbp2 and Akt signaling (Fig. 2A). Analysis of publicly available RPPA data in melanoma patient-derived xenograft (PDX) models (34) identified a direct correlation between IGFBP2 protein expression and phosphorylation (S473 and T308) of AKT (Fig. 2B and C). To confirm that this increase in p-AKT was mediated by IGFBP2, we cultured human melanoma cells with young CM and young CM with recombinant IGFBP2 (rIGFBP2). Cells treated with rIGFBP2 increased their levels of IGFBP2 and key downstream signaling markers such as p-AKT (Fig. 2D). As we have previously published, when exposed to aged versus young fibroblasts, melanoma cells increase lipid accumulation, as demonstrated by BODIPY staining (5).

To determine the contribution of IGFBP2 to this accumulation, we knocked-down IGFBP2 in aged fibroblasts (Fig. 2E), and then exposed melanoma cells to CM from the control and IGFBP2 knockdown fibroblasts. We found that knockdown of IGFBP2 in the aged fibroblasts decreased BODIPY staining in melanoma cells (Fig. 2F). Treatment of young fibroblasts with rIGFBP2 and subsequent treatment with the CM derived from that experiment increased the levels of BODIPY staining in the melanoma cells (Fig. 2G). We performed qRT-PCR on melanoma cells cultured with young and aged CM to determine whether treatment with CM caused an increase in transcription of *IGFBP2* in melanoma cells. There does not appear to be to be an increase in the transcription of *IGFBP2* (Fig. 2H), suggesting that IGFBP2 is taken up by melanoma cells rather than induced. We overexpressed IGFBP2 in melanoma cells (Fig. 2I) and observed increased p-AKT signaling and increased BODIPY staining in melanoma cells (Fig. 2J).

**Figure 3:**
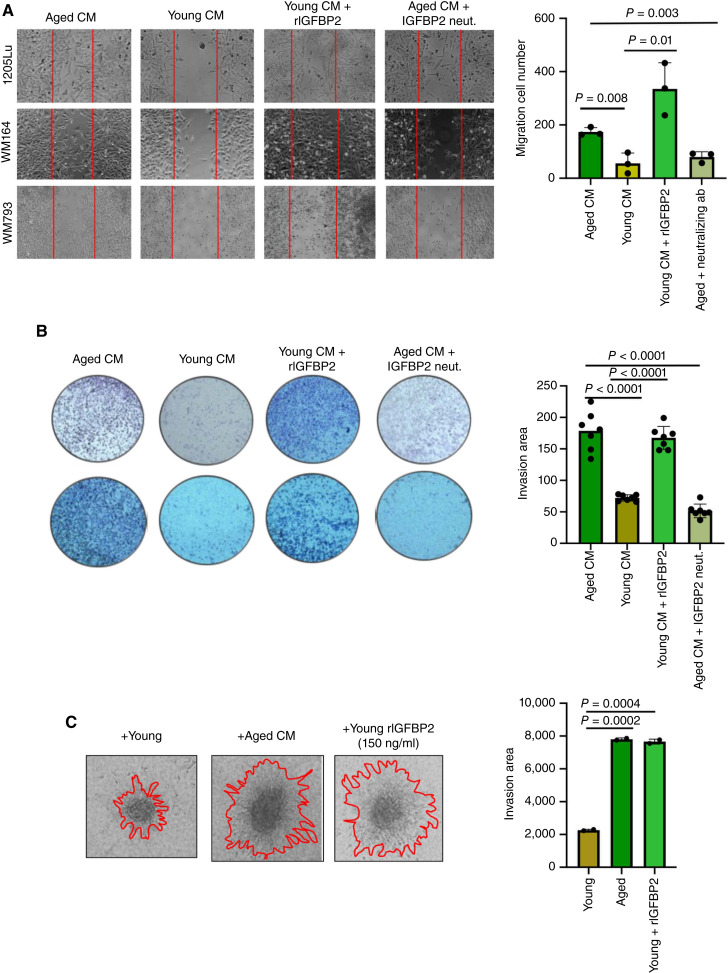
IGFBP2 increases melanoma cell migration and invasion. **A,** Wound healing assay of human melanoma cells (1205Lu, WM164) cultured with young or aged CM in the presence or absence of a neutralizing IGFBP2 antibody (5 mg/mL) or recombinant IGFBP2 (150 ng/mL). **B,** Matrigel invasion assay of melanoma cells (1205Lu, WM164) cultured with young and aged CM in the presence or absence of a neutralizing IGFBP2 antibody or recombinant IGFBP2. **C,** Melanoma cells (1205Lu) grown in 3D spheroids cultured with aged CM and young CM in the presence or absence of recombinant IGFBP2 (150 ng/mL) for 48 hours. GraphPad Prism 8 was used for plotting graphs and statistical analysis. Unpaired *t* test was performed.

Previous data from our laboratory have indicated that melanoma cells increase their invasion in response to CM from aged fibroblasts (4). We next wanted to observe if lipids would promote invasion in melanoma cells. We showed that adding lipids, such as Albumax, a lipid-rich cocktail, can significantly increase invasion of melanoma cells (Supplementary Fig. S2A). To test whether IGFBP2 in an aged versus young environment played a role in melanoma cell invasion, we first performed 2D wound healing assays. Wound healing assay analysis showed us that aged fibroblast CM increased melanoma cell migration over young CM, and that this could be reversed using a neutralizing antibody against IGFBP2, and similarly, the impact of young CM on melanoma cell migration could be increased using a recombinant IGFBP2 (Fig. 3A). We then performed a Boyden chamber assay to test for invasion and observed a similar result (Fig. 3B). We then performed a 3D spheroid assay, in which we embedded melanoma cell spheroids in collagen with young or aged fibroblasts, in which IGFBP2 was modulated, i.e., rIGFBP2 in young fibroblasts (Fig. 3C; Supplementary Fig. S2B) or neutralizing IGFBP2 antibody in aged fibroblasts (Fig. 3C; Supplementary Fig. S2C). In all of these experiments, aged fibroblasts were able to increase invasion of melanoma cells but less so in the absence of IGFBP2, whereas rIGFBP2 treatment or IGFBP2 overexpression in melanoma cells was able to increase invasion of melanoma cells in both young CM or in the presence of young fibroblasts.

**Figure 4:**
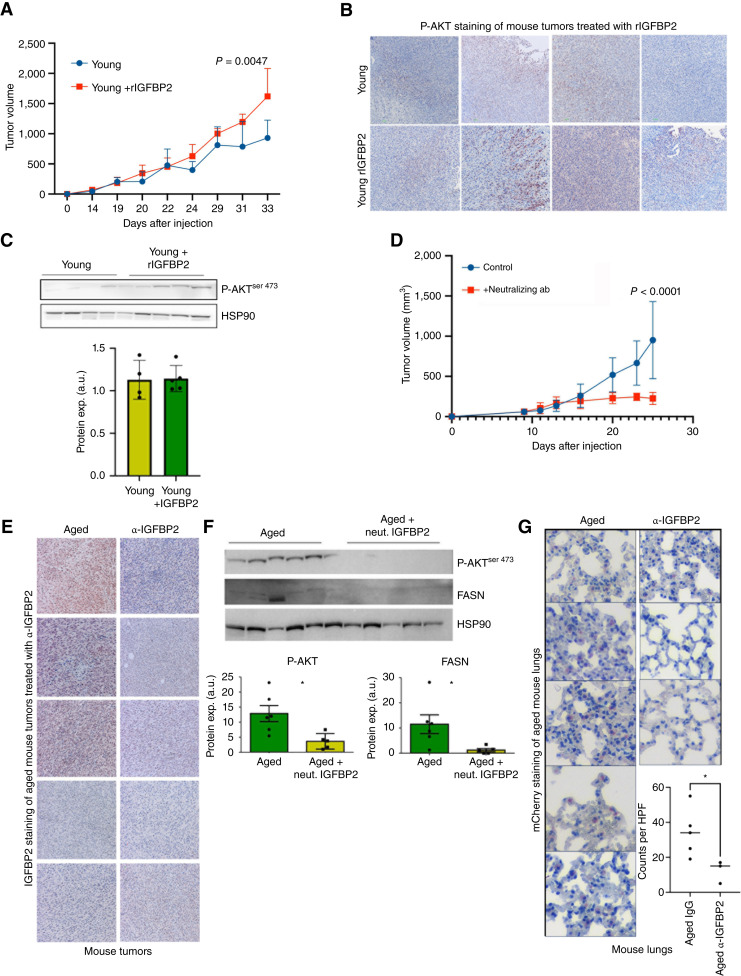
Igfbp2 increases melanoma tumor growth and metastasis *in vivo*. **A,** mCherry-tagged YUMM1.7 murine melanoma cells were grown in young (8 weeks old) C57BL6 mice. Tumor growth of young mice after i.p. treatment with 500 ng recombinant Igfbp2 or PBS (5 mice/group, treated i.p., 2 times a week, after tumors were palpable). **B,** IHC analysis of Igfbp2 in tumors from young mice treated with PBS or recombinant Igfbp2. **C,** Western blot analysis of tumor lysate from young mice treated with PBS and recombinant Igfbp2. Densitometry of phospho-AKT relative to HSP90 loading control. **D,** Tumor growth curve of YUMM1.7 melanoma cells subdermally injected in old (52 weeks old) mice treated i.p. with Igfbp2-neutralizing antibody (at a concentration of 1 mg/kg every day, *n* = 5) vs. an IgG control (*n* = 5). **E,** IHC analysis of Igfbp2 staining in aged mice treated with either an IgG control or neutralizing Igfbp2 antibody. **F,** Protein expression analysis was performed on tumor lysate from aged mice treated with IgG or neutralizing Igfbp2 antibody. Quantification analysis of phospho-Akt and Fasn immunoblotting relative to Hsp90 loading control. **G,** Analysis of mCherry-positive cells in lungs of tumor-bearing aged mice treated with a neutralizing Igfbp2 antibody or IgG control. *, *P* < 0.05 student *t* test was used. GraphPad Prism 8 was used for plotting graphs and statistical analysis.

Finally, we wanted to test the impact of modulating Igfbp2 in young and aged mice *in vivo*. We first examined the impact of Igfbp2 modulation on the growth of mCherry-tagged YUMM1.7 melanoma cells *in vivo*. As observed in other cancers, recombinant Igfbp2 increased the growth of tumors in young mice (Fig. 4A; refs. 35, 36). We confirmed moderate increases in Igfbp2 expression in the tumors of young mice treated with rIgfbp2 (Fig. 4B). We next examined tumor lysates by Western blot analysis for downstream markers of Igfbp2 signaling. We found that rIgfbp2 increased p-Akt in the tumors of young mice (Fig. 4C). We then tested whether inhibiting Igfbp2 signaling in aged mice could impact tumor growth. Treatment with a neutralizing antibody against Igfbp2 decreased tumor growth in aged mice (Fig. 4D). IHC of primary tumors confirmed the decrease in Igfbp2 expression upon treatment with the neutralizing antibody (Fig. 4E). Additionally, Western blot analysis of tumor lysates from aged mice treated with anti-Igfbp2 showed decreases in Fasn and p-Akt levels (Fig. 4F). Finally, we examined the lungs of the aged mice treated with IgG or anti-Igfbp2 for metastasis using an anti-mCherry antibody to detect melanoma cells in the lung. We found that neutralizing Igfbp2 resulted in fewer metastases from the primary site (Fig. 4G; Supplementary Fig. S3). Overall, our results show that Igfbp2 plays a role in driving melanoma metastasis and growth in an age-specific manner and that neutralizing Igfbp2 levels in mice decreases tumor growth and lung colonization.

## Discussion

Older patients with melanoma display more aggressive disease relative to their younger counterparts, as evidenced by a higher metastatic burden in distant organs and worse survival outcomes. Despite the advances in targeted and immune-based melanoma treatment strategies, most elderly patients still succumb to their disease due to their metastatic burden. Metabolic plasticity is required to allow cancer cells to survive the nutritionally diverse environments they encounter as they locally invade, enter the circulation, and ultimately leave the circulation to colonize distant organs (13, 37, 38). Notably, the metabolic landscape that tumor cells encounter radically changes during organismal aging, which has significant implications on tumor biology and phenotypes (39, 40). Our previous data demonstrate that host stromal fibroblasts exhibit marked metabolic reprogramming and alterations in their lipid secretion profile during organismal aging, translating to adaptive changes in melanoma cell uptake and utilization of aged fibroblast-derived lipids in the aged tumor microenvironment (TME) to escape therapy (5). In agreement, it was recently reported that serum isolated from elderly patients containing by-products of methylmalonic acid conferred chemoresistance in human triple-negative breast cancer and lung cancer (40). Nonetheless, our understanding of the dynamic relationship between organismal aging, metabolism, and tumor biology is only in its infancy.

Here, we report that in addition to elevated secretion of lipids capable of altering melanoma metabolism, aged stromal fibroblasts also secreted increased IGFBP2 levels that metabolically reprogram melanoma cells to synthesize lipid droplets that drive a pro-invasive/metastatic cell state. Lipid droplets serve essential physiologic roles in cell biology, signaling, membrane formation, and energy source; and lines of evidence show that tumor cells also leverage lipids to drive aggressive phenotypes (41, 42). Lipid droplets are composed mainly of triglycerides, cholesterol ester surrounded with a phospholipid monolayer which plays a role not only as a source of energy but also in cell membrane formation and signaling, which are used by cells to proliferate, generate cell protrusions, and invade (43–45). Other studies have shown that melanoma, breast cancer, and ovarian cancer cells, among others, can uptake lipids from adipocytes to increase their proliferation and invasion (10, 46, 47). Our data are consistent with recent reports in other cancers that reveal the importance of IGFBP2 in tumor growth and metastasis (36, 48, 49), and we identify aged stromal fibroblasts as a significant source of IGFBP2 in the aged TME.

In line with what has been observed in the senescence field, in which senescent cells secrete high levels of IGFBPs, we observe an increase in the secretion of IGFBP2 in aged fibroblasts (50, 51). The role of IGFBPs in tumor progression and therapy resistance varies by cancer type. IGFBP2 overexpression occurs in advanced cancers, including ovarian cancer, prostate cancer, and glioblastoma, and its high expression has been linked to an aggressive phenotype (36, 48, 49). Our data show that aged fibroblasts secrete elevated levels of IGFBP2, which elevates the metastatic capacity of melanoma cells.

Mechanistically, our studies suggest that increased IGFBP2 leads to elevated lipid synthesis by melanoma cells. Exogenous administration of rIgfbp2 *in vivo* increased p-AKT levels in tumors implanted in young mice. Notably, treating aged mice with Igfbp2-neutralizing antibody decreases p-AKT^ser473^ and FASN expression. Moreover, treating young mice with rIgfbp2 increased tumor growth, whereas neutralizing Igfbp2 decreased tumor growth. Our study demonstrates the importance of the aged TME in driving tumor progression and highlights the need for therapeutic interventions guided by the age of the patients that prevents tumor metastasis. These data provide the rationale that IGFBP2 may serve as a tractable therapeutic target to address elevated metastatic burden of aged patients with melanoma.

## Supplementary Material

Supplemental Figure 1 Overview of IGFBP2 expression in patient data bases, additional pAKT staining, and oil red O zoom out.

Supplemental Figure 2 Supplemental Figure 2 shows melanoma cells treated with Albumax, as well as an additional cell line showing the contribution of IGFBP2 to invasion

Supplemental Figure 3 Supp Fig 3 shows the zoom -out of Figure 4, to show a larger field of view for staining of mCherry cells
