# Supplementary material for: Age-Related Increases in IGFBP2 Increase Melanoma Cell Invasion and Lipid Synthesis
Source: Cancer Res Commun. 2024 Aug 2;4(8):1908–18. doi: 10.1158/2767-9764.CRC-23-0176 (PMC11295880; doi:10.1158/2767-9764.CRC-23-0176)
Supplement: Supplemental Figure 1 — Overview of IGFBP2 expression in patient data bases, additional pAKT staining, and oil red O zoom out. [file crc-23-0176_supplemental_figure_1_supps1.pdf]

Supplemental Figure 1

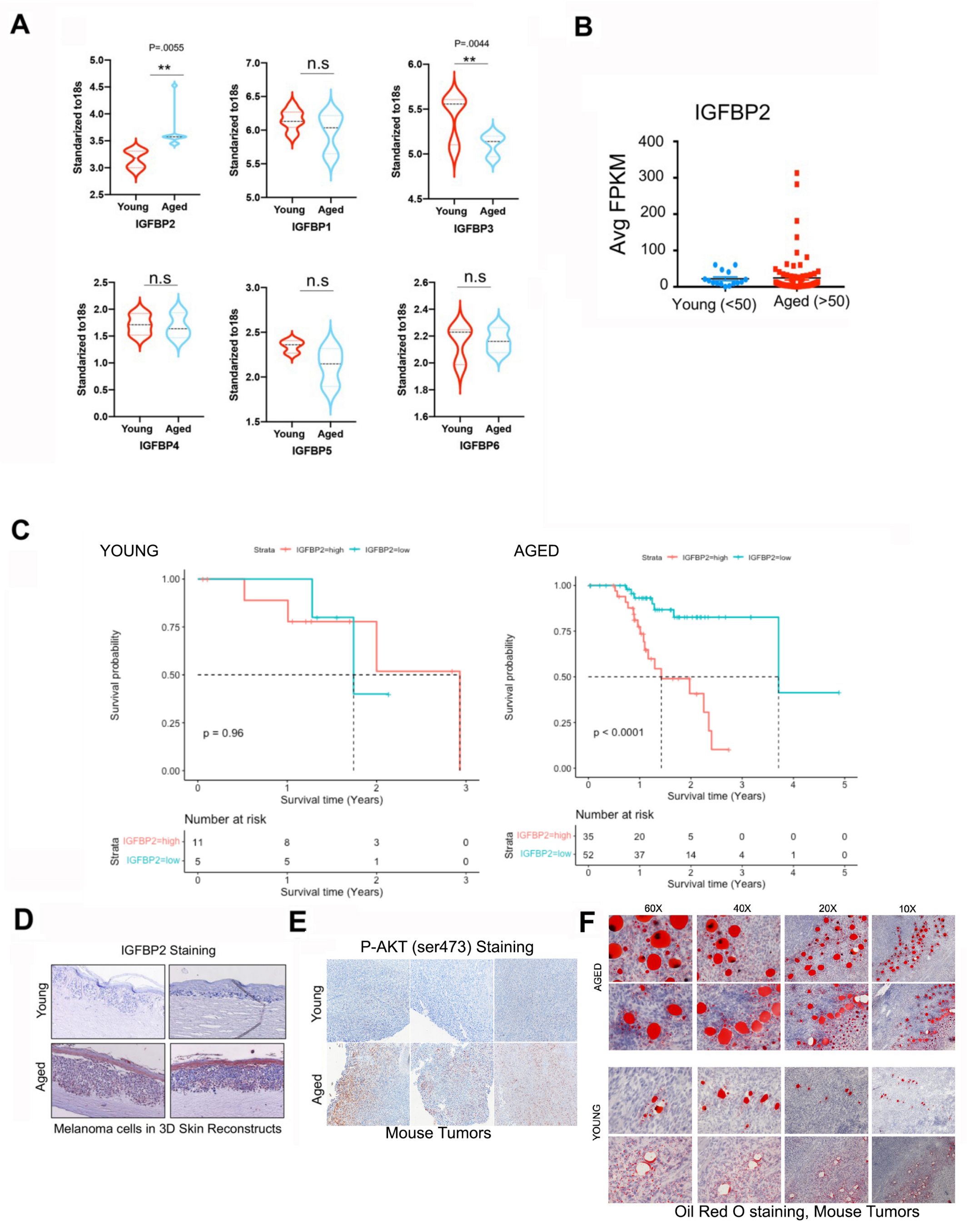

Supplemental Figure 1. Aged fibroblasts and melanoma tumors in aged microenvironments have high expression of IGFBP2. A) RT-PCR analysis of IGFBP1-6 in aged dermal fibroblasts. B) Analysis of IGFBP2 expression in melanoma from the human protein atlas. C) Kaplan Meyer plot survival relative to IGFBP2 expression in melanoma patient tumor samples according to age. Human Protein Atlas analysis set the FPKM cutoff to 10.62, and a p value of 0.00024. D) IHC of IGFBP2 staining in young and aged human skin reconstructs. E) Additional samples showing IHC of P-AKT (Ser473) staining in tumors from young and aged mice. F) Oil Red O staining of tumors from young and aged mice- additional magnifications of the tumors shown in Figure 1H.
