# Supplementary material for: Age-Related Increases in IGFBP2 Increase Melanoma Cell Invasion and Lipid Synthesis
Source: Cancer Res Commun. 2024 Aug 2;4(8):1908–18. doi: 10.1158/2767-9764.CRC-23-0176 (PMC11295880; doi:10.1158/2767-9764.CRC-23-0176)
Supplement: Supplemental Figure 2 — shows melanoma cells treated with Albumax, as well as an additional cell line showing the contribution of IGFBP2 to invasion [file crc-23-0176_suppl.figure_2_supps2.pdf]

Supplemental Figure 2

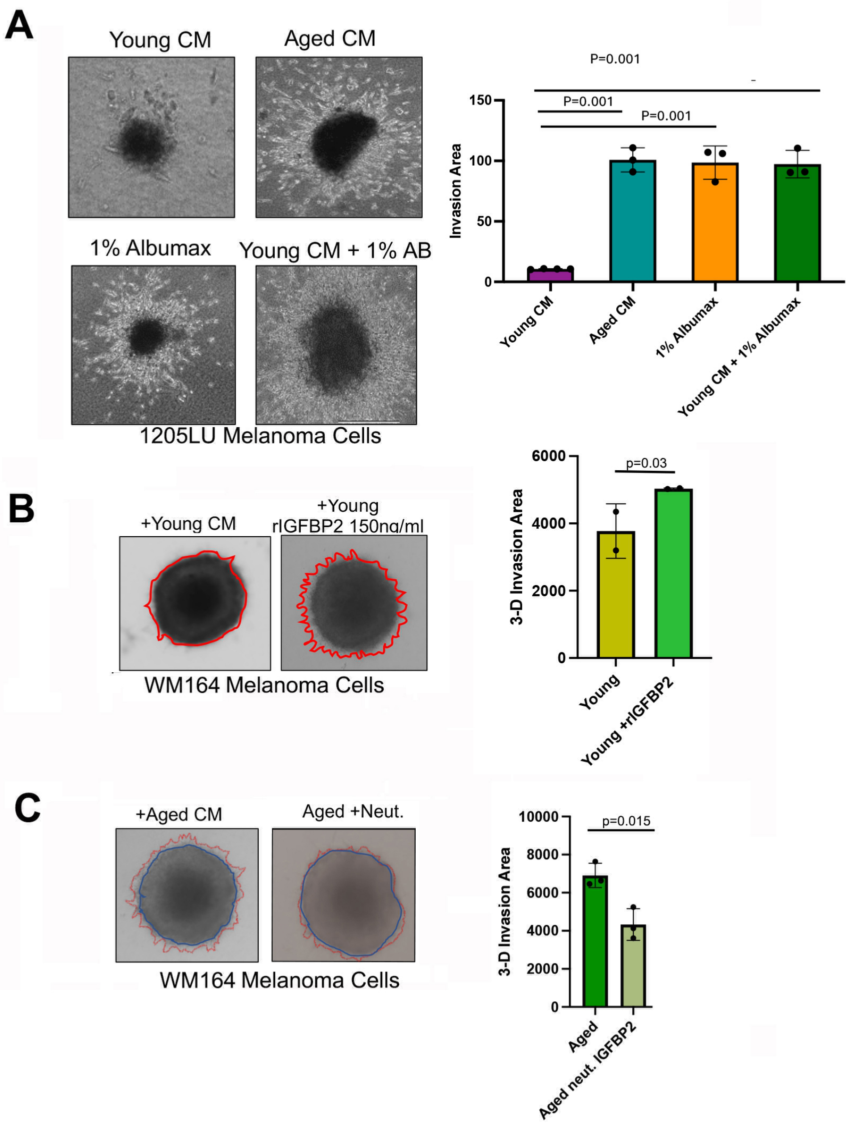

Supplemental Figure 2. IGFBP2 treatment promotes invasion. A) Melanoma cells (1205Lu) grown in 3D spheroids cultured with young fibroblast conditioned media in the presence or absence of 1% Albumax, aged fibroblast conditioned media, or regular culture media with 1% Albumax for 48 hrs. B) Melanoma cells (WM164) grown in 3D spheroid assay cultured with young fibroblast conditioned media in the presence or absence of 150ng/mL recombinant IGFBP2. C) Melanoma cells (WM164) grown in 3D spheroid assay cultured with aged fibroblast conditioned media in the presence of either an IgG control or a neutralizing IGFBP2 antibody (5µg/mL) for 48 hrs. GraphPad Prism 8 was used for plotting graphs and statistical analysis. Significance was designated as follows: **P* < 0.05; ***P* < 0.01; ****P* < 0.001; *****P* < 0.0001, unpaired t-test was performed.
