# Supplementary material for: Age-Related Increases in IGFBP2 Increase Melanoma Cell Invasion and Lipid Synthesis
Source: Cancer Res Commun. 2024 Aug 2;4(8):1908–18. doi: 10.1158/2767-9764.CRC-23-0176 (PMC11295880; doi:10.1158/2767-9764.CRC-23-0176)
Supplement: Supplemental Figure 3 — Supp Fig 3 shows the zoom -out of Figure 4, to show a larger field of view for staining of mCherry cells [file crc-23-0176_supplemental_figure_3_supps3.pdf]

Supplemental Figure 3

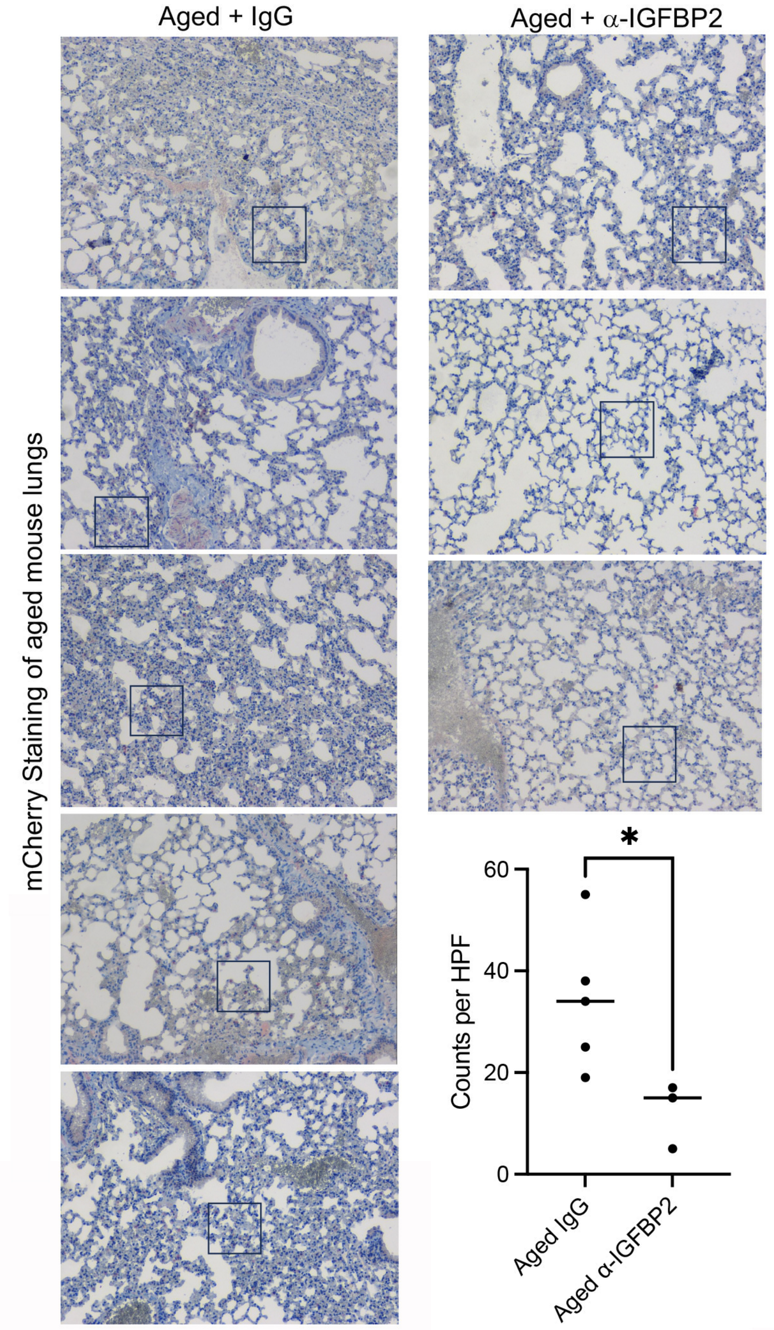

Supplemental Figure 3. Inhibiting IGFBP2 reduces metastasis. Analysis of mCherry positive cells in lungs of tumor bearing aged mice treated with a neutralizing IGFBP2 antibody or IgG control, larger fields of view of images from Figure 4 (boxes indicate areas imaged for Figure 4). * Indicates $p<0.05$ student t-test was used.
